# Platelet Hemostasis Reactions at Different Temperatures Correlate with Intracellular Calcium Concentration

**DOI:** 10.3390/ijms231810667

**Published:** 2022-09-14

**Authors:** Igor Mindukshev, Ekaterina Fock, Irina Dobrylko, Julia Sudnitsyna, Stepan Gambaryan, Mikhail A. Panteleev

**Affiliations:** 1 Sechenov Institute of Evolutionary Physiology and Biochemistry, Russian Academy of Sciences, 44 Thorez Ave., 194223 Saint Petersburg, Russia; 2 Center for Theoretical Problems of Physicochemical Pharmacology, Russian Academy of Sciences, 30 Srednyaya Kalitnikovskaya St., 109029 Moscow, Russia

**Keywords:** ADP, platelet, shape change, aggregation, clot reaction, intracellular calcium, laser diffraction, fluorescence analysis

## Abstract

Hypo- and hyperthermia affect both primary and secondary hemostasis; however, there are controversial data concerning platelet activation and the underlying mechanisms under hypo- and hyperthermia. The discrepancies in the data could be partly explained by different approaches to hemostatic reactions analysis. We applied a new LaSca-TMF laser particle analyzer for a simultaneous fluorescence and laser scattering analysis of platelet responses at different temperatures. Human platelets were activated by ADP in a wide range of temperatures, and platelet transformations (e.g., a shape change reaction, aggregation and clot formation) and the intracellular calcium concentration ([Ca^2+^]_i_) were analyzed by LaSca-TMF and confocal microscopy. The platelet shape change reaction gradually increased with a rising temperature. The platelet aggregation strongly decreased at low ADP concentrations with the augmentation of the temperature and was independent of the temperature at high ADP concentrations. In contrast, the clotting time decreased with a temperature increase. Similar to the aggregation response, a rise in [Ca^2+^]_i_ triggered by low ADP concentrations was higher under hypothermic conditions and the differences were independent of the temperature at high ADP concentrations. We showed that the key reactions of cellular hemostasis are differentially regulated by temperature and demonstrated for the first time that an accelerated aggregation under hypothermic conditions directly correlated with an increased level in [Ca^2+^]_i_ in platelets.

## 1. Introduction

The regulation of temperature is involved in the control of reactions in most fields of life including the reactions that are responsible for thrombosis and hemostasis. A temperature of 37.5 °C is believed to be the optimal temperature to maintain proper hemostatic balance, and changes in body temperature could strongly influence the coagulation system and platelet function [1,2]. Generally, low temperatures slow the protein–protein interactions and enzymatic reactions down, whereas at higher temperatures, the internal energy of the reacting molecules increases; however, the outcome is not obvious because inhibitory reactions are likewise slowed down. At high temperatures, nevertheless, the increased internal energy of the molecules could also lead to the breakdown of the enzyme structure as a consequence of disrupted intra- and intermolecular bonds [3]. Hypo- and hyperthermia affect both the primary and secondary hemostatic systems. Inhibition of the coagulation system and clotting time decrease in fibrin network formation during hypothermia and an increase in coagulation during hyperthermia are well documented in clinical and experimental animal studies [3,4,5]. At the same time, there are controversial data on the effects of different temperatures on primary hemostasis, particularly platelet activity. For example, platelets have been shown to be hyperactive at both low [6,7,8,9,10,11] and high temperatures [12,13]. Additionally, a prolonged bleeding time during surgeries in hypothermic conditions [14,15,16] and platelet hypo- [17,18] and hyperreactivity [1] in hyperthermic conditions have been reported. 

The described diversity of platelet reactivity in hypo-and hyperthermic conditions could be explained, in part, by the different methodological approaches used for platelet functional tests. Platelets are the key players controlling hemostasis, and they are the main triggers of the thrombus formation; therefore, the analysis of platelet responses to different temperatures is very important for understanding the molecular mechanisms responsible for the adaptation of platelets to hypo- and hyperthermic conditions. Different methods for aggregation assessment, including the classical Born method of light transmission, impedance aggregometry of whole blood, and others, are commonly used for the analysis of platelet function (for review see [19]); however, these methods have some limitations, mainly related to the calcium concentration in whole blood and platelet-rich plasma (PRP) samples, which depends on the use of extracellular calcium-lowering anticoagulants such as citrate, EGTA, or EDTA. A low extracellular calcium concentration significantly affects the intracellular calcium response, which in turn strongly determines most platelet transformation reactions, including a shape change [20,21], granule release, aggregation, and activation of the coagulation cascade [22,23,24,25]. To overcome these problems, we upgraded the recently introduced LaSca laser analyzer based on the laser diffraction method (low angle light scattering) [26] for a platelet transformation analysis in diluted PRP with a physiological extracellular calcium concentration (2 mM). This method was previously successfully used for the characterization of platelet purinergic (P2Y1, P2Y12, and P2X1) receptors [26], analysis of cGMP/PKG [27], and cAMP/PKA [28] inhibitory systems in platelets. In our new device, we introduced a fluorescence channel that allows, in addition to the characterization of the platelet shape change reaction and aggregation, a simultaneous registration of intracellular calcium concentration ([Ca^2+^]_i_) changes in dynamics. In addition, for the analysis of the coagulation system, we included an algorithm for a clot formation description. 

For the investigation of temperature regulation of platelet responses, we challenged platelets by ADP stimulation in different conditions (17–41 °C) and analyzed the responses using confocal microscopy and laser diffraction methods. We showed that the key reactions of cellular hemostasis are dependent and differentially regulated by temperature. Under hypothermic conditions, the velocity of the shape change reaction and clot formation decreased, whereas the aggregation was accelerated at low (<600 nM) doses of ADP. An accelerated aggregation directly correlated with the [Ca^2+^]_i_, which was the highest at low temperatures (25 °C). 

## 2. Results

### 2.1. Description of the Upgraded Laser Diffraction Method for the Registration of a Platelet Shape Change Reaction, Aggregation, and Clot Formation

**Figure 1:**
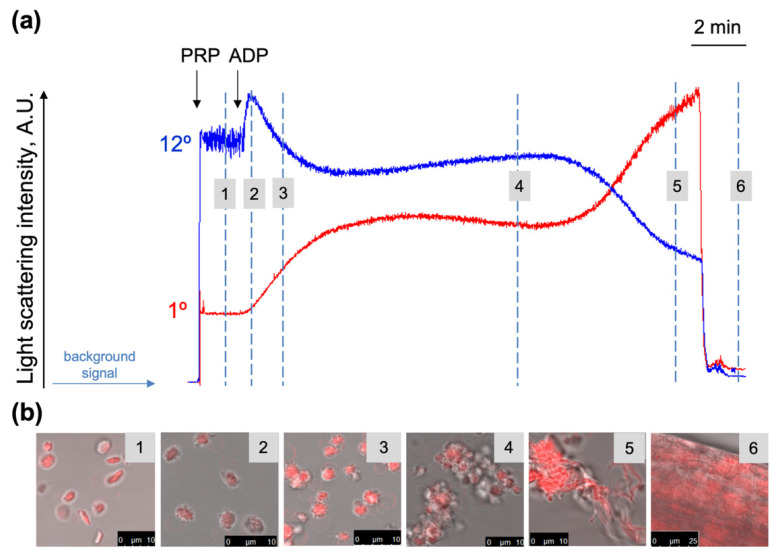
Registration of platelet transformations (shape change, aggregation, and clot formation) by laser diffraction method. (**a**) PRP diluted in the HEPES buffer (1 × 10^7^ platelets/mL) was added to the cuvette with continuous stirring (1200 rpm) at 25 °C. After 2 min of incubation, ADP (800 nM) was added, and light scattering intensity (LSI) was monitored for the scattering angles of 1° and 12°. For better visualization, the LSI signal at 12° was amplified by 50; (**b**) at each step of platelet transformation, samples were withdrawn from the LaSca cuvette, fixed with formaldehyde (2%), and then stained with Nile Red (3 µg/mL, 10 min, RT) for confocal microscopy analysis of platelet aggregation and clot formation (Photos 1–6). Shown are the original representative traces and photos from four independent experiments.

The basic principles of the laser diffraction method for the evaluation of platelet activation were introduced previously (Mindukshev et al., 2012) and for a more detailed description of the method see the Supplementary Materials (Section S1). Here, the updated method is presented in brief, including the proper temperature control, platelet disaggregation characterization, and clot formation. Additionally, in the new device, we introduced a fluorescence channel for the simultaneous measurement of the platelet shape change, aggregation, and intracellular calcium concentration changes in kinetics. The corresponding platelet transformation reactions were confirmed microscopically (Figure 1b). An initial rapid reaction (during less than 30 s) to ADP (0.8 µM), measured as LSI signal changes at 12°, was caused by a platelet shape change, i.e., a platelet transformation from a discoid to spherical form and pseudopodia formation (Figure 1b, photos 1, 2). The inception of the aggregation process was registered as the increase in the LSI signal at 1° and the decrease at 12°, which correspond to the formation of the first platelet aggregates consisting of 2–4 platelets (Figure 1b, photo 3). The next step of the platelet aggregation was characterized by a dramatic increase in the number of platelets involved in the aggregate formation (Figure 1b, photo 4). During this process, the LSI at 1° and at 12° did not significantly change and the signal stayed at the plateau for approximately 10 min (Figure 1a, the area between points three and four). The next step was characterized by the initiation of clot formation (Figure 1b, photo 5) and the corresponding LSI signal at 1° was increasing and the signal at 12° was decreasing for 3–4 min (Figure 1a, the area between the points four and five). At the last stage, all the platelets formed a single clot with fibrin fibers on the surface of the magnetic steer (Figure 1b, photo 6), the solution in the cuvette became transparent and both LSI signals at 1° and at 12° returned to the background level (Figure 1a, the area between the points five and six). 

**Figure 2:**
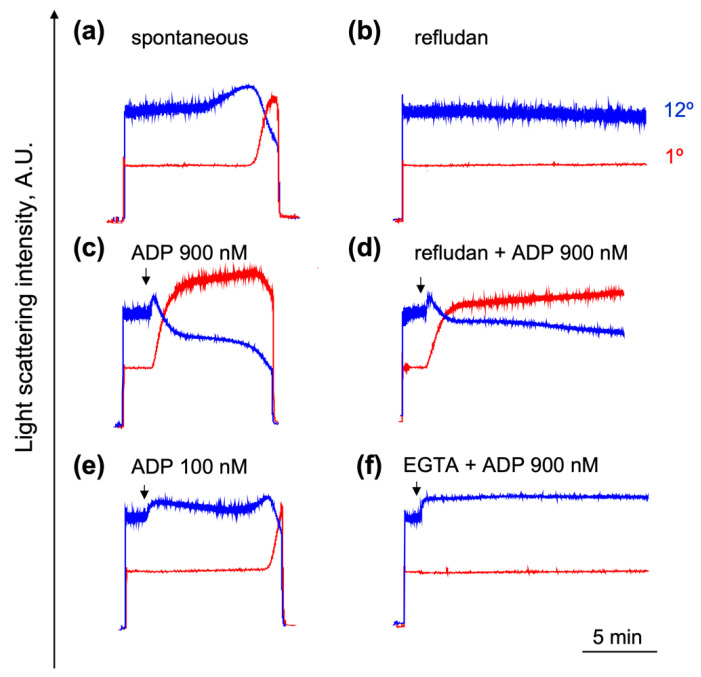
Characterization of the clot formation reaction by the laser diffraction method. PRP diluted in the HEPES buffer (1 × 10^7^ platelets/mL) was added to the cuvette with a continuous stirring (1200 rpm) at 25 °C. The clot formation reaction was characterized as a returning of LSI at both angles to the background level. (**a**) Spontaneous clot formation accompanied by a slowly developing (slow increase in LSI at 12°) shape change reaction; (**b**) Refludan (20 µg/mL) completely prevented the reaction of clot formation; (**c**) platelet activation by ADP (900 nM) slightly accelerated clot formation reaction; (**d**) Refludan inhibited clot formation but had no effect on the shape change reaction and aggregation; (**e**) induction of the shape change reaction only by low (100 nM) ADP had no significant effect on the time of clot formation; (**f**) clot formation and aggregation, but not the shape change, induced by ADP (900 nM), were inhibited in calcium-free (2 mM EGTA) media. Presented are the original traces out of four independent experiments.

**Figure 3:**
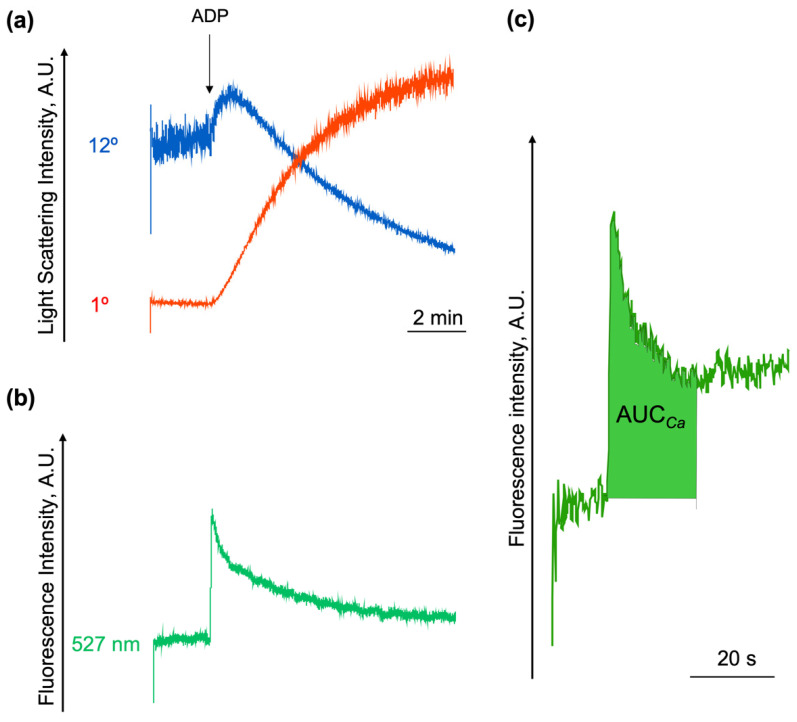
Simultaneous registration of platelet transformation and changes in [Ca^2+^]_i_. PRP was loaded with Fluo-3 AM (5 µM), then diluted in HEPES buffer (1 × 10^7^ platelets/mL) and added to the cuvette with continuous stirring (1200 rpm) at 25 °C. After the registration of the basal signal (3 min), ADP (800 nM) was added to the platelet suspension. (**a**) The platelet shape change reaction and aggregation were monitored as in Figure 1 and Figure 2 according to the changes of LSI signal measured at 12° and 1°, correspondingly, and (**b**) [Ca^2+^]_i_ signal was detected at FL1 channel; (**c**) to characterize ADP-induced [Ca^2+^]_i_ changes in dynamics, the AUC*_Ca_* within the first 20 s after the application of ADP was calculated. Raw traces from the LaSca laser analyzer, shown are the data from one experiment out of four.

During disaggregation, the LSI signals measured at 1° and 12° were contradirectional (Figure 1, the area between points four and five; Figure 2). The platelet disaggregation process strictly depended on the agonist concentration and temperature (Figure 2). The registration of the clot formation reaction by the laser diffraction was not characterized previously [26]; therefore, here we present a detailed analysis of this reaction. In our experimental conditions, a spontaneous, i.e., without platelet stimulation by agonists, clot formation was observed after 12 ± 3 min of platelet suspension stirring in the presence of 2 mM of calcium at 25 °C. Refludan completely prevented the reaction by inhibiting thrombin generation, and the LSI signals remained at the initial level for at least 40 additional min (Figure 2a,b). Platelet activation by a high (900 nM) ADP concentration induced a shape change and aggregation reaction and slightly reduced the time to clot formation (10 ± 3 min). Refludan, in this case, again completely prevented clot formation, but not the shape change and aggregation (Figure 2c,d). When the platelets were stimulated by a low (100 nM) ADP concentration, which induced only a shape change reaction without aggregation, the clot formation time did not differ significantly from the spontaneous reaction and was inhibited by the Refludan also (Figure 2e). Clot formation and platelet aggregation are strictly dependent on extracellular calcium; however, in the presence of EGTA, only a shape change was initiated and both of the platelet reactions were inhibited (Figure 3f).

### 2.2. Adaptation of the LaSca-TMF Laser Particle Analyzer for the Evaluation of the Intracellular Calcium Concentration 

To monitor the [Ca^2+^]_i_, we upgraded our laser analyzer and introduced a 488 nm laser with a fluorescence detector supplied by a 527 nm filter (FL1). This upgraded device allows the simultaneous registration of platelet transformations (e.g., shape change and aggregation) together with changes in [Ca^2+^]_i_ in dynamics. The platelet shape change reaction and aggregation were detected according to the changes in the LSI measured at 1° and 12° as described previously (Section 2.1 of the manuscript), and the changes in [Ca^2+^]_i_ were registered at the FL1 channel according to Fluo-3 fluorescence (Figure 3). To prove that our method was adequate for a [Ca^2+^]_i_ assessment, we calibrated [Ca^2+^]_i_ in silent platelets stained by Fluo-3 (Supplementary Material, Section S2) and showed it to be around 100 nM (Supplementary Figure S5), which was in agreement with the literature [29,30,31,32,33].

The increase in [Ca^2+^]_i_ occurred immediately (during the first 1–2 s) after the application of ADP and gradually decreased during 20 s. For the future analysis of [Ca^2+^]_i_ changes, we used an accepted parameter for the characterization of [Ca^2+^]_i_ mobilization, namely, the area under the curve (AUC*_Ca_*) within a 20 s interval after an agonist application [34,35,36].

### 2.3. Changes in Temperature Differentially Affected the Platelet Transformation 

**Figure 4:**
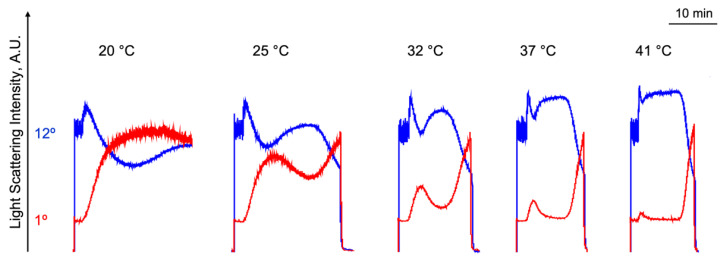
ADP-induced platelet transformation at different temperatures. PRP diluted in HEPES buffer (1 × 10^7^ platelets/mL) was added to the cuvette with a continuous stirring (1200 rpm) at 25 °C. After 2 min of incubation, ADP (300 nM) was added and dynamical changes in light scattering intensity were registered for the scattering angles of 1° and 12° at 20 °C, 25 °C, 32 °C, 37 °C, and 41 °C. For better visualization, the data of LSI at 12° were multiplied by 50. Shown are the original representative traces from four independent experiments.

In the literature, there is still no consensus on how hypo- or hyperthermia affects platelet transformation and blood coagulation. The discrepancies in the data could be partly explained by the assessment of different platelet reactions (e.g., aggregation, integrin activation, granule release, platelet adhesion, etc.), different temperatures (mild or profound hypo/hyperthermia), or different methods for the analysis of the blood coagulation system [19]. In this study, we analyzed platelet transformations (e.g., the shape change reaction, aggregation, and clot formation) in response to different ADP concentrations and in the range of temperatures from 20 °C to 41 °C. In Figure 4, the summarized results of the platelet transformations triggered by 300 nM of ADP at different temperatures are presented. First, the rapid shape change reaction started at all the tested temperatures (for the details, see the next section). The amplitude of the platelet aggregation gradually decreased with a rise in temperature (Figure 4, red traces) which resulted from the opposing process of disaggregation (a simultaneous decline in the LSI signal at 1° and increment at 12°) velocity accrual (Figure 4). Interestingly, the clot formation reaction progressed in the direction opposite to that of the aggregation. The time of clot formation gradually decreased with an increasing temperature (Figure 4). Platelet responses to temperature are complex and include all phases (e.g., shape change reaction, aggregation and clot formation); therefore, we focus below on each of these reactions in detail.

### 2.4. The Velocity of Shape Change Accelerated with a Rise in Temperature

**Figure 5:**
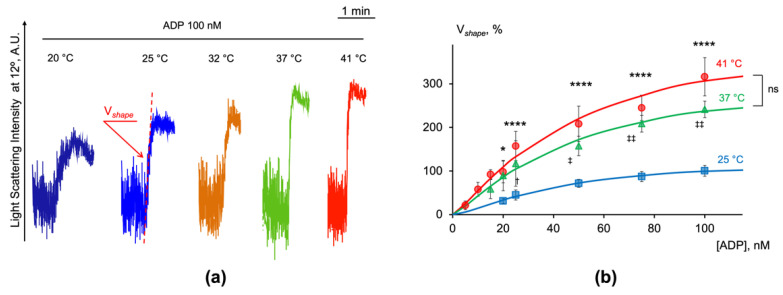
The velocity of shape change reaction gradually increased with the rise in temperature. PRP diluted in HEPES buffer (1 × 10^7^ platelets/mL) was added to the cuvette with continuous stirring (1200 rpm) at indicated temperatures. After 2 min of incubation, ADP was added in the concentration range from 5 to 100 nM and the dynamics of the LSI signal changes was monitored at 12°. (**a**) Shown are the original representative traces from four independent experiments; (**b**) the dose-response curves of shape change velocity plotted against ADP concentrations at 25°, 37°, and 41 °C. Points indicate the normalized V*shape* data, continuous lines represent fits of the Hill equation to the experimental data. Data are presented as means ± SD, one-way ANOVA, Tukey post-hoc, for 41 °C compared to 25 °C: *, *p* < 0.05, ****, *p* < 0.0001; for 37 °C compared to 25 °C: ^†^, *p* < 0.05, ^‡^, *p* = 0.003, ^‡‡^, *p* < 0.0001; ns, not significant; *n* = 4.

With the LaSca-TMF device, we were able to detect a platelet shape change starting from a very low dose (5 nM) of ADP (Supplementary Figure S1) and with a maximal velocity achieved at 100 nM of ADP. The amplitude and velocity of the shape change reaction gradually increased with the rising temperature under ADP stimulation (Figure 5a). The velocity of the shape change reaction (V*shape*) was calculated by the original LaSca v.1498 software. To characterize the shape change reaction and compare the velocity at different temperatures, we analyzed the platelet responses triggered by ADP in the range from 5 to 100 nM and calculated the V*shape* for each. The V*shape* triggered by 100 nM of ADP at 25 °C was taken as 100% (Figure 5b). Then, the normalized V*shape* values were plotted against the ADP concentration and analyzed. 

The dose-response curves of the V*shape* against the ADP were saturable; therefore, the maximal velocity of the shape change when the system was saturated (V*shape*_max_), the concentration of ADP which triggered the half-maximum effect (EC_50_), and the Hill coefficient (h) were calculated according to the Hill equation (Table 1). Additionally, the half-reaction time (τ_1/2_) for the ADP (100 nM) was calculated at different temperatures. The increase in temperature from 25 °C to 37 °C accelerated the V*shape*_max_ from 126.25 ± 13.77 to 327.51 ± 19.09 (Table 1); therefore, the temperature coefficient of the reaction (Q_10_) being greater than 2, indicated that the reaction can involve changes in the protein conformation [37,38,39]. The sensitivity of the V*shape* to the agonist concentration did not vary significantly and rested between 40 and 50 nM (Table 1, EC50). The Hill coefficient value was close to 1, indicating that the V*shape* change conformed to the Michaelis–Menten model, and not Hill–Langmuir [40].

The data presented in Table 1 clearly demonstrate that the V*shape*_max_ gradually increased, and the τ_1/2_ gradually decreased with the rise in temperature, while the EC_50_ and the Hill coefficient did not change significantly (Table 1). 

### 2.5. Hypothermia Accelerated Platelet Aggregation

**Figure 6:**
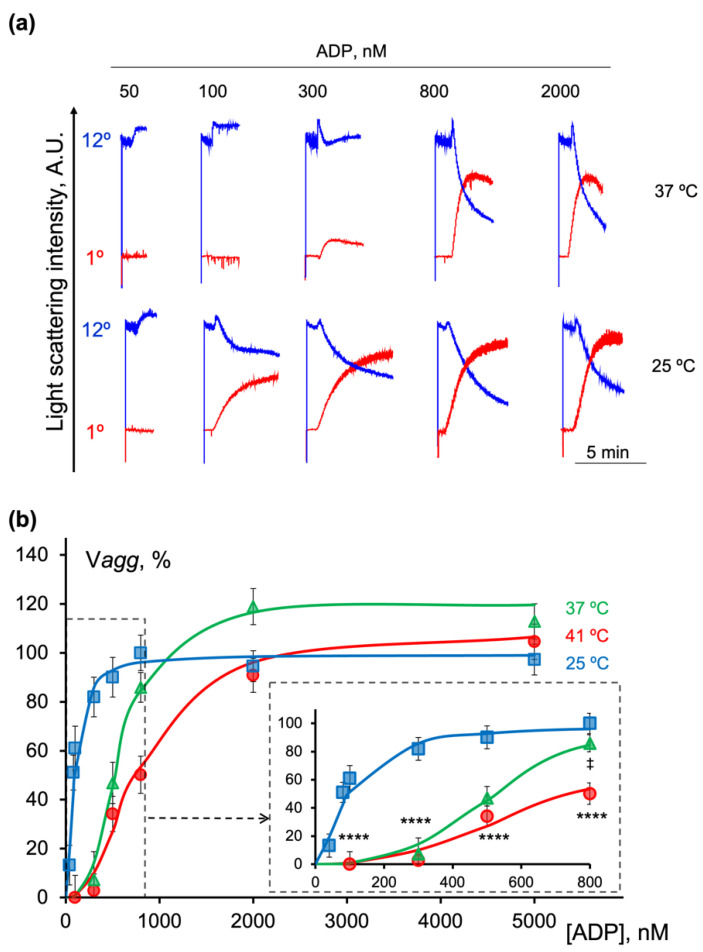
Initiation of platelet aggregation and its velocity were differentially regulated by temperature. PRP diluted in HEPES buffer (1 × 10^7^ platelets/mL) was added to the cuvette with a continuous stirring (1200 rpm) at the indicated temperature. After 2 min of incubation, indicated concentrations of ADP were added and the LSI signal was monitored in dynamics at 1° and 12°. For better visualization, the data of LSI at 12° were multiplied by 50. (**a**) Shown are the original representative traces from four independent experiments; (**b**) the dose-response curve of aggregation velocity (V*agg*) plotted against ADP concentrations. Points indicate the normalized V*agg* data, continuous lines represent fits of the Hill equation to the experimental data. The enlarged part of the graph of the initial steps of aggregation is shown in a separate box. Data are presented as means ± SD, two-way ANOVA, Tukey’s post-hoc; **** *p* < 0.0001, ^‡^, *p* = 0.0075 compared to 25 °C, *n* = 6.

As shown in Figure 4, the platelet aggregation declined with the temperature increase in response to 300 nM of ADP. Here, we present a more detailed analysis of the temperature-dependent alterations of platelet aggregation. At 37 °C, 50 and 100 nM of ADP induced only a shape change reaction, and the aggregation manifested at 300 nM with the gradual increase in response intensity (Figure 6a, blue traces corresponding to 37 °C). At 25 °C, 50 nM of ADP did not trigger the aggregation, while at the same time, 100 nM of ADP led to a pronounced response which increased dose-dependently (Figure 6a, red traces corresponding to 25 °C). Interestingly, at higher ADP concentrations (800 and 2000 nM), the initial velocity of aggregation (V*agg*) accelerated with the rise in temperature (Figure 6a, red traces, 6b). The maximum effect of ADP on the V*agg* was reached at 2000 nM (37 °C and 41 °C) and at 25 °C it was already reached at 800 nM of ADP (Figure 6a). To compare the data, the V*agg* triggered by 800 nM of ADP at 25 °C was taken as 100%. The normalized V*agg* data were then plotted against the agonist concentration to obtain the dose-response curve for further analysis (Figure 6b). The calculated EC_50_ for an initial aggregation velocity at 25 °C (104 ± 16 nM) was significantly lower than that at 37 °C (599 ± 76 nM) and 41 °C (873 ± 61 nM) (Figure 6b, Table 2). The dose-response curves of the initial velocity of the aggregation at all tested temperatures were characterized by saturation (Figure 6b); however, it can be clearly seen that for the higher temperatures (37 °C and 41 °C), more agonist was needed to trigger the response (Figure 6b, graph for low ADP concentrations).

### 2.6. Hypothermia Increased the Clotting Time

The reaction of a clot formation is an important characteristic of the coagulation system. In our experimental conditions at 25 °C, spontaneous clots formed approximately after 15 min of continuous stirring even without any agonists (Figure 2). We analyzed the time to clot formation for spontaneous and ADP-induced (2 µM) clotting at 25 °C, 37 °C, and 41 °C. Platelet activation by the ADP did not significantly change the clotting time compared to the non-stimulated platelets. In both cases, the time to clot formation decreased with the rise in the temperature (Table 3).

### 2.7. [Ca^2+^]_i_ Decreased with the Rise in Temperature 

In ADP-stimulated platelets, the dynamics of [Ca^2+^]_i_ is characterized by two opposing processes including a fast increase in [Ca^2+^]_i_, mediated mainly by activation of the IP3/IP3R system, and its gradual decrease mediated by SERCA, which pumps calcium back to the stores, and PMCA responsible for calcium efflux from the cells [41,42,43].

**Figure 7:**
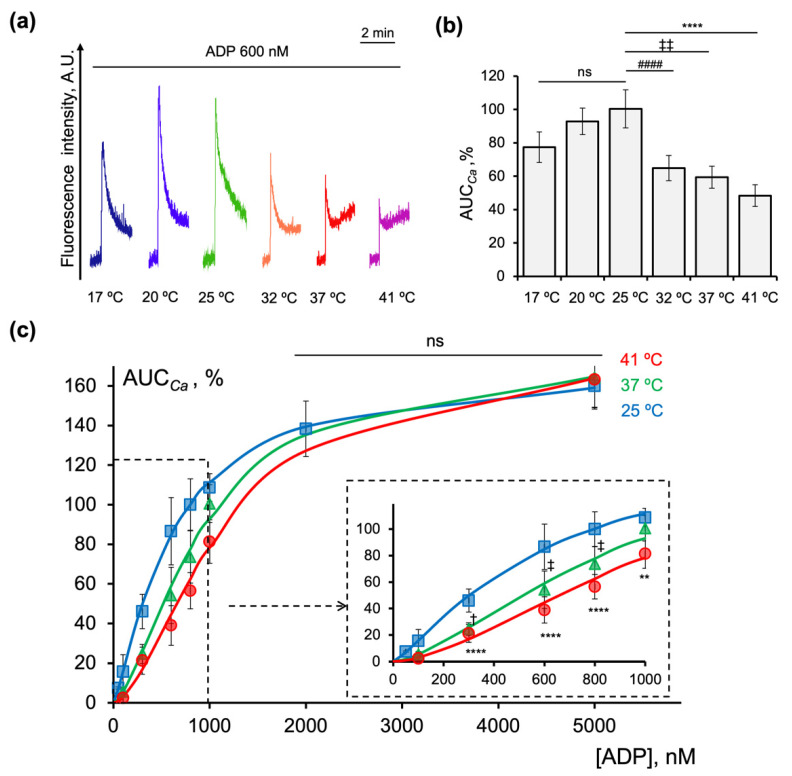
[Ca^2+^]_i_ decreased significantly with the temperature rise. PRP was stained with Fluo-3 AM (5 µM, 30 min, RT), diluted in HEPES buffer (1 × 10^7^ platelets/mL), and added to the cuvette with continuous stirring (1200 rpm) at indicated temperatures. After 2 min of incubation, ADP (600 nM) was added, and [Ca^2+^]_i_ was assessed in dynamics according to the Fluo-3 fluorescence registered at FL1 (527 nM). (**a**) Representative traces from four independent experiments; (**b**) quantification of the data presented in (**a**); (**c**) dose-response curves of AUC*_Ca_* plotted against ADP concentration. For all calculations, the AUC*_Ca_* for 800 nM ADP-stimulated platelets at 25 °C was taken as 100%. Points indicate the normalized AUC*_Ca_* data, and continuous lines represent fits of the Hill equation to the experimental data. The dose-dependency of AUC*_Ca_* at the low concentrations of ADP (from 5 to 1000 nM) is emphasized in the separate box. Data are presented as means ± SD, *n* = 8; for (**b**) one-way ANOVA, Tukey’s post-hoc; ns: not significant; ****, *p* < 0.0001 (25 °C vs. 41 °C), ^‡‡^, *p* < 0.0001 (25 °C vs. 37 °C), ^####^, *p* < 0.0001 (25 °C vs. 32 °C). For (**c**) two-way ANOVA, Tukey’s post-hoc; ^†^, *p* < 0.05, ^‡^, *p* < 0.0001 (37 °C vs. 25 °C); **, *p* = 0.0063, ****, *p* < 0.0005 (41 °C vs. 25 °C).

The initial velocity of aggregation was analyzed 20 s after the ADP administration; therefore, the changes in [Ca^2+^]_i_ were also calculated as the area under the curve (AUC*_Ca_*) measured within the first 20 s after the agonist application (Supplementary Figure S5). We evaluated [Ca^2+^]_i_ in platelets stimulated by 600 nM of ADP at different temperatures (Figure 7a) and calculated the corresponding AUC*_Ca_* (Figure 7b). The [Ca^2+^]_i_ decreased significantly with the rise in temperature, whereas at the lower temperatures (from 17 °C to 25 °C), there were no significant differences detected except a slight increment in AUC*_Ca_*. Next, we compared the AUC*_Ca_* triggered by ADP in the range from 50 nM to 5 μM at different temperatures (Figure 7c). The AUC*_Ca_* induced by 800 nM of ADP at 25 °C was taken as 100%. The dose-dependency curves for all the tested temperatures were characterized by saturation; therefore, as previously noted, we used the Hill model to describe them quantitatively (Table 4). It can be clearly seen that at low ADP concentrations [Ca^2+^]_i_ at 25 °C, less agonist was needed to activate the response (Figure 7b, separate graph), and these data directly correlated with the data on the V*agg*, which was also higher in hypothermic conditions only at low ADP concentrations (compare Figure 6 and Figure 7). At high ADP concentrations (up to 5 µM), there were no significant differences in [Ca^2+^]_i_ in relation to the different temperatures (Figure 7b).

## 3. Discussion

Hemostasis is a complex process that includes primary hemostasis (platelet activation) and secondary hemostasis (activation of the coagulation system). Both primary and secondary hemostasis are highly sensitive to changes in the environmental and body temperature, and severe disorders of this system could occur in hyper-and hypothermia and could subsequently cause a coagulopathy leading to hemorrhages or thrombotic events [1,2,44,45]. Nevertheless, hyper- and hypothermia are used in several clinical settings such as cooling the patient, which is routinely applied in cardiac surgery to protect the organs against ischemia [46]. Hypothermia commonly occurs in severe trauma patients, being one of the components of the “trauma triad” along with coagulopathy and acidosis [2,47,48]. Platelet concentrate storage is also highly dependent on temperature, thus, hypothermia of different degrees, RT or cold storage is used [8,49,50]. Local hyperthermia is used as an adjuvant therapy in several cancer types [51,52]. Moreover, heatstroke and viral infection-induced fever often induce multi-organ dysfunction connected with hemorrhages as a result of coagulation system activation and thrombocytopenia [53,54].

In the literature devoted to the regulation of hemostasis at different temperatures, the accepted consensus is that hypothermia accelerates platelet activation, whereas the coagulation system is more sensitive to hyperthermia [3]. For example, hypothermia increases platelet activation and aggregation [8,55,56], whereas coagulation is attenuated by hypothermia [44,45,57], but the molecular mechanisms of hypothermia-induced platelet activation are not yet fully clear. It was previously shown that ADP played a key role in hypothermia-induced platelet activation [8,10] and that hypothermia enhanced platelet aggregation at low and medium ADP doses, while at high ADP doses (10–20 µM) the effects were minor or even absent [6,7,11]. Additionally, platelet αIIbβ3 integrin activation and P-selectin surface exposure were enhanced under hypothermia; however, the dose-dependence patterns for aggregation [6,11,56], integrin activation, and granule release [6,56] were different. These differences were not related to changes in the αIIbβ3 or GPIbα expression, and hypothermia had no effects on vWF binding to platelets [10,58]. Moreover, the exposure of platelets to 20 °C for 10 min resulted in αIIbβ3 integrin activation [55], which was suppressed by the PI3K p110β inhibitor alone or in combination with P(2)Y blockers [58]. At the same time, the activity of the P2Y12 receptor evaluated by PGE1-induced VASP phosphorylation levels, with or without an additional ADP stimulation, was not affected during storage [59]. Similarly, an ADP-induced inhibition of VASP phosphorylation in platelets from healthy volunteers was not affected by hypothermia in either the presence or absence of clopidogrel [7].

In this study, we applied our recently developed and upgraded LaSca-TMF laser analyzer for the analysis of platelet transformations in a wide range of temperatures (20–41 °C) with a special emphasis on the molecular mechanisms that could be involved in the modulation of temperature-dependent changes in platelet reactivity. Two major mechanisms responsible for platelet transformations include intracellular calcium mobilization, which increases in response to an agonist stimulation, and activation of the cyclic nucleotide pathways (PKA and PKG), which represents the major inhibitory pathways in platelets (from the literature). We confirmed the results of [6,7,11], namely, that the acceleration of ADP-induced platelet aggregation was detected only at low (up to 600 nM) ADP concentrations. At the same time, for higher ADP concentrations (1–6 µM), the maximal initial velocity of aggregation at 37 °C was even higher than that at 25 °C (Figure 6, Table 2). These data strictly correlated with the dynamics of [Ca^2+^]_i_ changes and showed that AUC*_Ca_* decreased significantly with temperature increase at low (600 nM) ADP concentrations (Figure 7a), and that these differences were abolished at high (1–6 µM) ADP concentrations (Figure 7b, Table 3). Thus, our data are in good agreement with the well-known fact that the degree of platelet activation/aggregation is directly connected with the [Ca^2+^]_i_ concentration [22,23,24,25]. To evaluate whether the differences in platelet reactivity at 37 °C and 25 °C were related to the activation of PKA/PKG, we analyzed the VASP phosphorylation, which is often used as a marker of these kinase activations [60,61], in platelets under these conditions (Supplementary Figure S6). We evaluated the VASP phosphorylation by the flow cytometry and Western blot methods. Both methods did not demonstrate any changes in the VASP phosphorylation associated with the incubation of platelets at different temperatures (Supplementary Figure S6).

The simultaneous registration of platelet transformations, including the shape change reaction, aggregation, [Ca^2+^]_i_ dynamics, and clot formation under the same constant conditions, is one of the advantages of the upgraded LaSca-TMF analyzer. We showed for the first time that the shape change reaction, in contrast to the aggregation and [Ca^2+^]_i_ dynamics, was enhanced with a rise in temperature (Figure 5, Table 1). The molecular mechanisms of the accelerated shape change reaction in hyperthermia are not clear yet and merit future examinations. In agreement with the literature, using the LaSca-TMF analyzer, we also detected an acceleration in the clot formation with a temperature increase [62,63].

In summary, we showed that the key reactions of cellular hemostasis are highly dependent and differentially regulated by temperature. Under hypothermic conditions at low doses of ADP, the velocity of shape change reaction and clot formation decreased, whereas the aggregation accelerated. Additionally, an accelerated aggregation directly correlated with the free calcium concentration in platelets and was independent of the PKA/PKG activity, the main inhibitory pathways in platelets.

## 4. Materials and Methods

### 4.1. Study Design

- Ethics approval. This study was approved by the Ethics Committee of the Sechenov Institute of Evolutionary Physiology and Biochemistry of the Russian Academy of Sciences (protocols no. 3–03 from 2 March 2021, and no. 1–04 from 7 April 2022) and was performed in compliance with the Declaration of Helsinki. All participants gave written informed consent before inclusion into the study.
- Population. Healthy randomly selected volunteers (*n* = 15, 7 males, 8 females, and 21–55 years) were enrolled at the Laboratory of Cellular mechanisms of blood homeostasis of the Sechenov Institute of Evolutionary Physiology and Biochemistry of the Russian Academy of Sciences, between December 2021 and May 2022. The exclusion criteria were pregnancy, breastfeeding, participation in another clinical trial, any form of medication interfering with coagulation, and known coagulopathies of the participant.

### 4.2. Reagents and Working Buffers

To evaluate the effects of temperature on the platelet function, the isotonic HEPES buffer (in mM: NaCl, 150; KCl, 2; MgCl_2_, 1; Glucose, 5; HEPES, 10; pH 7.4; 300 mOsm kg H_2_O) was used. An amount of 2 mM Ca^2+^, or 2 mM EGTA were added where indicated. The osmolality of the buffer was monitored by the Osmomat 030 cryoscopic osmometer (Gonotec GmbH, Berlin, Germany). All the buffer components, ADP, and Nile Red were purchased from SigmaAldrich (Munich, Germany). The Fluo-3 AM and Pluronic F-127 were obtained from Invitrogen (Carlsbad, CA, USA). The Refludan (lepirudin, recombinant hirudin) was from Celgene (Windsor, UK).

### 4.3. Platelet Preparation

Human platelets were prepared as described previously [64]. In brief, blood was collected by caudal venipuncture in 9NC S-monovette tubes (Sarstedt, Nümbrecht, Germany) with the addition of EGTA (2 mM) and centrifuged at 300× *g* (8 min; room temperature, RT). The supernatant containing plasma and platelets (PRP) was collected and rested at RT until the experiment. The Medonic-M20 hematological counter (Boule Medical A.B., Stockholm, Sweden) was used to control the platelet count and parameters.

### 4.4. Laser Diffraction Analysis with Simultaneous Fluorescence Detection

To monitor changes in the platelet functional state and intracellular calcium concentration, the LaSca-TMF laser microparticle analyzer (BioMedSystems Ltd., Saint Petersburg, Russia) was used. The PRP was suspended in a HEPES buffer (with a final concentration of 1 × 10^7^ platelets/mL) and added to the cuvette with continuous stirring (1200 rpm) at the indicated temperatures. The laser diode-generated monochromatic light beam (488 nm) was then directed through the cuvette with a platelet suspension. The intensity of the light scattered by the cells was continuously detected by forward scattering by photodiodes at 28 different angles in the range of 0.1° to 12° angles.

The method was based on the Mie theory of scattered light properties, which states that the light scattering intensity measured at a specific angle depends on the volume of a particle and the difference in the refractive indices of the particle and the medium. Thus, the analysis of the scattered-by-particle light accurately provides information about the illuminated particle [26].

#### 4.4.1. Detection of Platelet Functional Indices

The platelet shape change was characterized by an increase in the LSI at the scattering angle of 12°. The platelet aggregation was characterized by the LSI increase at the scattering angle of 1° with a simultaneous LSI decrease at the scattering angle of 12°. The initial velocity of aggregation was assessed 20 s after the agonist supplementation. The terminal stage of clot formation was characterized by the LSI decrease throughout the scattering angles. For a detailed description see Section 2.1.

#### 4.4.2. Analysis of Platelet Ca^2+^ Mobilization

To monitor the changes in [Ca^2+^]_i_, the PRP was incubated with Fluo-3 AM dye (5 μM) and Pluronic F-127 (0.02%) for 60 min at RT in the dark. Next, the PRP (with a final concentration of 1 × 10^7^ platelets/mL) was suspended in a HEPES buffer and analyzed simultaneously by both laser diffraction and fluorescent analysis. Intracellular Fluo-3 was excited at 488 nm and the emission was registered at 527 nm (FL1). For the calibration of the [Ca^2+^]_i_, platelets loaded with Fluo-3 AM were incubated with calcium ionophore A23187 (10 µM), and immediately after the maximal signal was achieved, EGTA (16 mM) was added, and the [Ca^2+^]_i_ calculated according to the standard equation (for details, see the Supplementary Section S2, or Supplementary Figure S5). Changes in the [Ca^2+^]_i_, were described as the dynamics of the fluorescence intensity (FI) measured at FL1 and as the area under the curve (AUC*_Ca_*) within 20 s after the application of the agonist. For a detailed description, see Section 2.2.

### 4.5. Confocal Microscopy Analysis of the Platelet Transformations 

The platelet suspension was withdrawn from the laser analyzer cuvette and immediately fixed by formaldehyde (2%) at the indicated stages of cell transformations. A Leica TCS SPII confocal microscope (Leica microsystems, Wetzlar, Germany) was used for the light and fluorescent microscopy of the fixed platelets. To visualize the cell membrane changes, the platelets were incubated with Nile Red (3 µg/mL, 10 min, RT) with the following dye excitation at 488 nm and emission registration in the wavelength range from 500 to 800 nm.

### 4.6. Hill Model

The data were analyzed and processed through the Hill equation according to [26] and additionally via GraphPad Prism v.9.

### 4.7. Data Analysis

The laser diffraction and fluorescent data were analyzed using the original software of the LaSca-TMF laser particle analyzer, LaSca v.1498 (BioMedSystems, Ltd., Saint Petersburg, Russia). For the Mie scattering approximation, the free MiePlot v.4.6 software was used [65]. The confocal microscopy data were processed and analyzed with the Leica TCS SPII confocal software (Leica Microsystems Heidelberg GmbH, Heidelberg, Germany). For the data analysis, GraphPad Prism v.9 (GraphPad Software Inc., San Diego, CA, USA) was applied. According to the Shapiro–Wilk’s test, the data were normally distributed; therefore, for group comparisons, a one-way or two-way ANOVA was used correspondingly with the appropriate post-hoc tests. One-sample or paired t-tests were used where applicable. Each dataset represents not less than three different experiments on the material taken from not less than three different healthy volunteers. Data are presented as the mean ± SD, and *p* < 0.05 was considered statistically significant.

## Figures and Tables

**Table 1:** Calculated values of the maximal velocity of platelet shape change (V*shape*_max_), EC_50_, Hill coefficient (h), and τ_1/2_ at 25 °C, 37 °C, and 41 °C.

Temperature	V*shape*_max_, %	EC_50_, nM	h	τ_1/2_, s
41 °C	435.00 ± 14.72 ****	49.44 ± 8.08	1.22 ± 0.07	4.60 ± 0.22 ****
37 °C	327.51 ± 19.09 ^‡‡^	46.44 ± 9.11	1.24 ± 0.09	6.10 ± 0.48 ^‡‡^
25 °C	126.25 ± 13.77	40.90 ± 3.48	1.45 ± 0.17	15.88 ± 1.71

The values were calculated from the experimental data presented in Figure 5b. Data are presented as means ± SD, one-way ANOVA, Tukey’s post-hoc; **** *p* < 0.0001, ^‡‡^, *p* < 0.0001 compared to 25 °C, *n* = 4.

**Table 2:** Calculated values of the maximal velocity of platelet aggregation (V*agg*_max_), EC_50_, and Hill coefficient (h) at 25 °C, 37 °C, and 41 °C.

Temperature	V*agg*_max_, AU	EC_50_, nM	h
41 °C	105.38 ± 13.12	873.68 ± 63.31 ****	2.70 ± 0.51 **
37 °C	120.20 ± 17.35 ^†^	599.29 ± 59.67 ^‡‡^	3.86 ± 0.41 ^‡‡^
25 °C	101.3 ±7.52	104.39 ± 11.96	1.79 ± 0.27

The values were calculated from the experimental data presented in Figure 6b. Data are presented as means ± SD; ^†^, *p* < 0.05, ^‡‡^, *p* < 0.0001, **, *p* = 0.0066, ****, *p* < 0.0001, compared with 25 °C, *n* = 6.

**Table 3:** The clotting time increased at lower temperatures.

Temperature	25 °C	37 °C	41 °C
Spontaneous clot formation, min	14.47 ± 2.40	9.81 ± 2.00 ^†^	9.51 ± 2.00 *
ADP-induced clot formation, min	13.30 ± 2.40	8.51 ± 1.63 ^†^	8.79 ± 2.90 *

Clot formation time calculated from the beginning until the signal from LSI at 1° and at 12° decreased dramatically (see Figure 2). Data are presented as means ± SD, one-way ANOVA, Tukey’s post-hoc; *, *p* < 0.05 (41 °C vs. 25 °C), ^†^, *p* < 0.05 (37 °C vs. 25 °C), *n* = 5–7.

**Table 4:** Calculated values of AUC*_Ca_*_max_, EC_50_, and Hill coefficient (h) for 25 °C, 37 °C, 41 °C.

Test Temperature	AUC*_Ca_* _max_, %	EC_50_ (Ca^2+^), nM	h (Ca^2+^)
41 °C	178.25 ± 12.45 ***	1156.00 ± 99.30 ****	1.67 ± 0.17 ***
37 °C	176.75 ± 11.15 ^‡^	939.20 ± 39.40 ^‡‡^	1.55 ± 0.09 ^‡^
25 °C	170.00 ± 8.00	603.30 ± 27.90	1.22 ± 0.07

The values were calculated from the experimental data presented in Figure 7. Data are presented as means ± SD, one-way ANOVA, Tukey’s post-hoc; ***, *p* < 0.0005, ****, *p* < 0.0001 (41 °C vs. 25 °C), ^‡^ *p* < 0.005, ^‡‡^, *p* < 0.0001 (37 °C vs. 25 °C), *n* = 4–6.

## Data Availability

The data underlying this article will be shared at reasonable request to the corresponding author.

## Supplementary Materials

The following supporting information can be downloaded at: https://www.mdpi.com/article/10.3390/ijms231810667/s1. Reference [66] is cited in the supplementary materials.
