# Supplementary material for: Platelet Hemostasis Reactions at Different Temperatures Correlate with Intracellular Calcium Concentration
Source: Int J Mol Sci. 2022 Sep 14;23(18):10667. doi: 10.3390/ijms231810667 (PMC9505593; doi:10.3390/ijms231810667)
Supplement: Supplementary file 1 [file ijms-23-10667-s001.zip › ijms-1892888-supplementary.pdf]

S1 Characterization of platelet transformation reactions by the laser diffraction method

S1.1 Parameters of shape change reaction

LaSca-TM laser particle analyzer allows simultaneous registration of light scattering intensity (LSI) at the scattering angles from 1° to 12° [1]. LSI at 12° was shown to be the most sensitive to the platelet shape change [1], therefore we chose it for platelet shape monitoring, and using microphotographs (Figure 1b) confirmed that the changes in LSI corresponded to the shape change reaction. For the quantitative characterization of shape change reaction, we introduced the V*shape* parameter, which is determined as the velocity of LSI increase measured at 12° within the first 10 s after application of ADP (Figure 1a).

Platelets are discoid plates with a high LSI noise level and during spherization, the noise level decreases. Correspondingly, the higher the noise level is, the wider the standard deviation of particle distribution by diameters, and vice versa. The laser diffraction method has already successfully been used by others [2] for the analysis of platelet shape change and it was shown that during this reaction, i.e., during the transition from the discoid to the spheroid shape, the oscillation amplitude (noise level) of light scattering intensity (LSI) signal decreased significantly. In our experiments, we confirmed this observation and showed that the basal noise level (2.6 ± 0.4) was significantly higher than the noise level after platelet activation (0.9 ± 0.5) (Supplementary Figure S1a). It should be mentioned that the newly upgraded laser analyzer (LaSca-TMF) enabled us to register the shape change reaction triggered by a very low (5 nM) ADP concentration (Supplementary Figure S1a). To model the platelet shape change reaction, we used the Mie scattering simulation program (MiePlot v.4.6 software developed by Philip Laven [3]). Thus, we assumed the control cells (which are approximately 3 μm in diameter) to be the particles with the wide (20%) distribution by size and compared them to the particles with the narrow (5%) distribution by size which reflect the cells that have undergone shape change, or spherization (Supplementary Figure S1b, inserted histogram). Next, we plotted the theoretical LSI obtained for both, control and ADP-stimulated platelets, against the scattering angle (angle diagram, or indicatrix; Supplementary Figure S1b). These calculations showed that at 8 -12° angles spherical particles with a standard deviation of 5% scatter with higher LSI than discoid cells with a standard deviation of 20% (Supplementary Figure S1b). Therefore, we calculated the ratio of LSI of the cells with 5% SD to 20% SD and plotted it against the scattering angles (Supplementary Figure S1c). Thus, it is clearly seen that at the scattering angles from 8 to 12° the shape change reaction could be accurately registered.

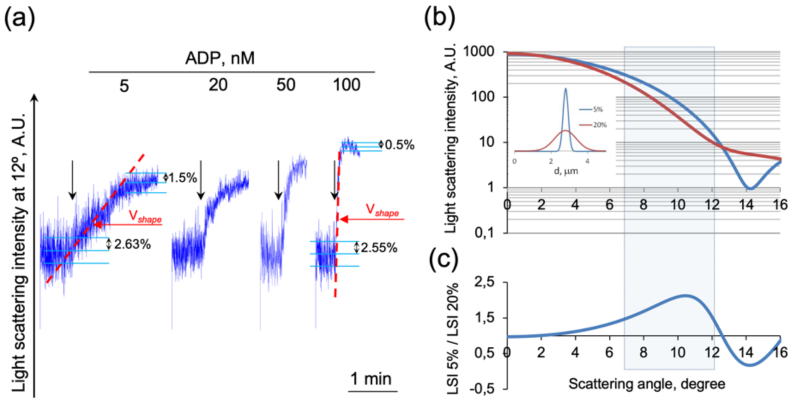

Supplementary Figure S1. Registration of platelet shape change reaction by laser diffraction method. PRP diluted in the HEPES buffer (1 x 10^7 platelets/ml) was added to the cuvette with a continuous stirring (1200 rpm) at 25°C. After 1 min of

incubation, the indicated concentrations of ADP were added and the changes in LSI measured at 12° were registered in dynamics. According to these data, the velocity of the shape change reaction (V*shape)* during the first 10 s after ADP stimulation could be calculated. **(a)** - The platelet shape change reaction triggered by ADP stimulation (5 – 100 nM). Shown are the representative traces from four independent experiments. (**b) –** Simulation of the angle diagram (indicatrix) for the 3 µm particles corresponding to control "discoid" platelets with high 20% SD (red traces) and particles corresponding to ADP-stimulated "spherical" platelets with low SD 5% (blue traces) plotted against the scattering angle (theoretical data obtained from MiePlot v.4.6 software [3]). The comparative histograms of the size distribution for the spherical particles with a diameter of 3 µm and SD 5% and SD 20% are shown in insertion. **(c) -** Ratio of indicatrix from (b) corresponding to 3 µm particles with SD 5% to 3 µm particles with SD 20% plotted against the scattering angle. The grey box marks the area of indicatrix that is most sensitive to the changes in SD for 3 µm particles.

S1.2 Identification of platelet aggregation by laser diffraction method

During the platelet aggregation process, we observed strong differences in LSI measured at different scattering angles [1]. To characterize this process properly we analyzed the ADP-stimulated platelets by confocal microscopy (Figure 1). We matched a set of curves obtained by LSI changes recorded at various angles (see below) with a microscopic analysis of the propagation of platelet aggregation in our model system. At the angles from 0.5 ° - 3° the LSI signal was characterized by an increase followed by a plateau, at 3° - 8° it increased in the beginning and then gradually decreased, whereas at the angles from 9 to 12° the signal had a rapid peak after which the signal only decreased (Supplementary Figure S2). According to the presented data we have chosen the LSI measured at 1 ° and 12 ° for characterization of the platelet aggregation process.

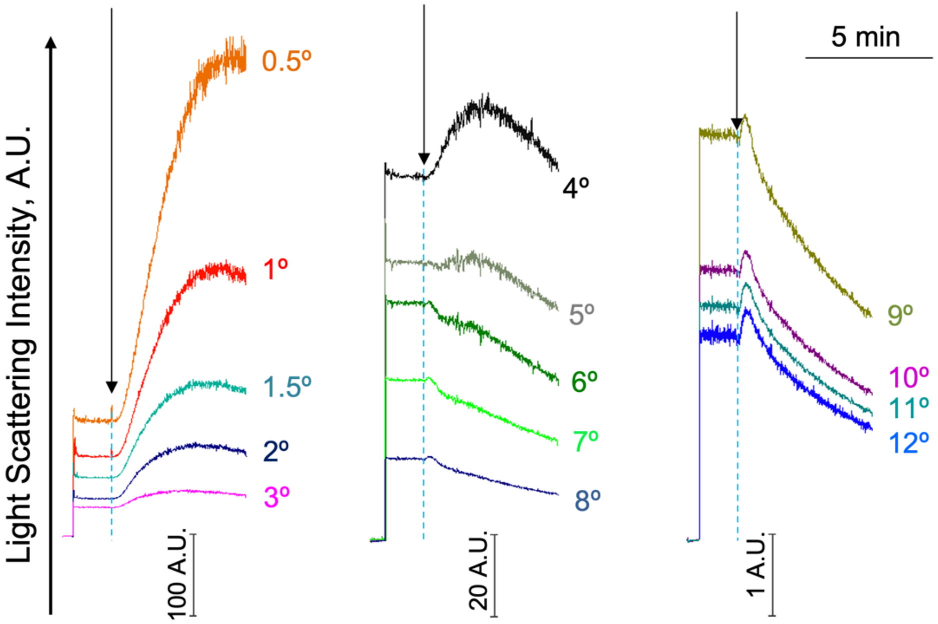

Supplementary Figure S2. Strong differences in LSI were measured at different scattering angles from a single ADP-stimulated platelet sample. The original records of LSI changes were detected at different scattering angles from ADP-stimulated platelets in dynamics. PRP diluted in the HEPES buffer (1 x 10^7 platelets/ml) was added to the cuvette with a continuous stirring (1200 rpm) at 25 °C. After 2 min of incubation, ADP (800 nM) was added and the LSI signal at the scattering angles from 0.5° to 12° was recorded in dynamics. The LSI data are divided into three groups of angles according

to the intensity of the signal. For better visualization, the LSI for the angles from 4° to 8° was multiplied by 5, for the angles from 9° to 12° - by 100. ADP addition is marked by black arrows.

Next, to describe the platelet aggregation at the different stages we analyzed platelets microscopically 10, 30, 60, 120, and 300 s after stimulation by ADP in indicated concentrations (Supplementary Figure S3a). According to the microphotographs cells/aggregates were ranked into 7 fractions containing: (1), single cells; (2) – (7), aggregates containing up to 5, 10, 30, 100, 200, and 500 platelets. Then the quantitative distribution of cells/aggregates was presented for 100 – 800 nM at 10 – 300 s (Supplementary Figure S3b). We showed that ADP at low (100 nM) concentrations induced the formation of small aggregates containing 2 – 5 cells mostly. ADP concentration increase triggered the gradual potentiation of the number of large aggregates (Supplementary Figure S3a, b).

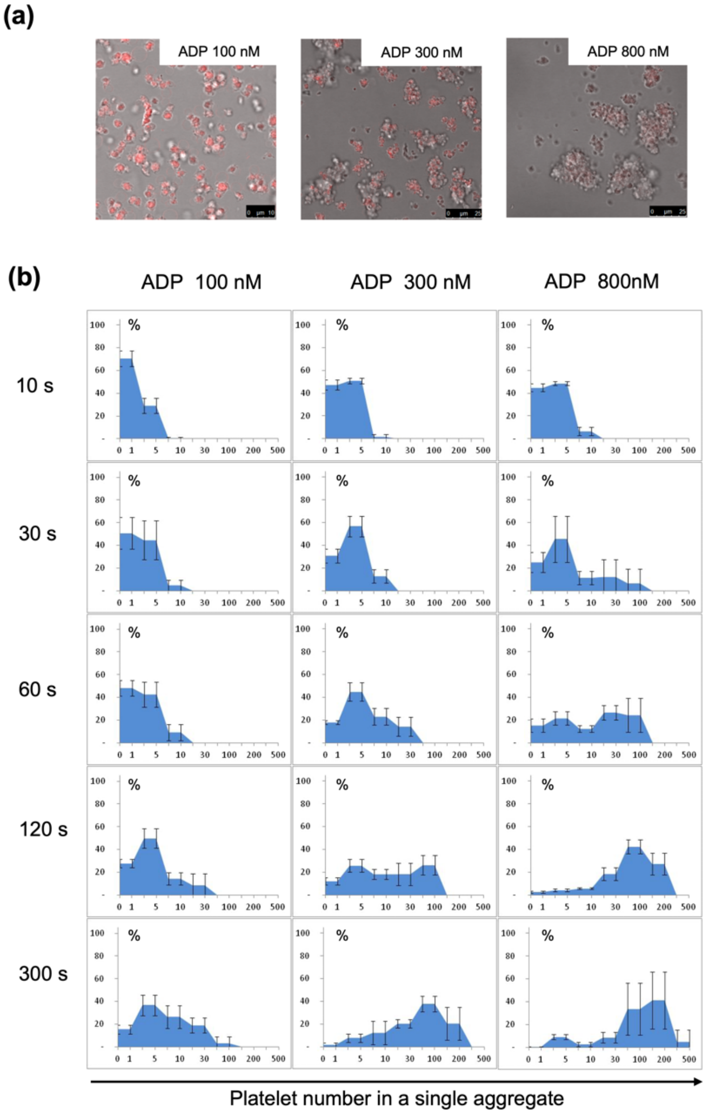

Supplementary Figure S3. Quantitative distribution of ADP-induced platelet aggregates by sizes. PRP diluted in HEPES buffer (1 x 10^7 platelets/ml) was added to the cuvette with a continuous stirring (1200 rpm) at 25ºC. After 2 min of incubation, ADP in indicated concentrations was added and after indicated time the samples of platelet suspensions were taken for quantitative analysis of aggregates by confocal imaging. Cells/aggregates were ranked into 7 fractions containing:

(1), single cells; (2) – (7), aggregates containing up to 5, 10, 30, 100, 200, 500 platelets. **(a) –** Representative microphotographs of platelet aggregates resulted from ADP stimulation in indicated concentrations for 120 s (corresponding to Supplementary Figure S3a, 120 s); **(b) –** Quantitative distributions of cells/aggregates formed after ADP stimulation, n = 4 for all calculations.

Based on the quantitative analysis of these data we developed a model for platelet aggregation characterization by laser diffraction method. Aggregation could be regarded as a process when the particle volume is increased with the consequent decrease of particle amount. Thus, the initial single spherical particles made up into dimers with a volume twice as much as the initial single particles and the number of particles correspondingly decreased two-fold with a consequent seven iterations. Then we calculated the diameters for the described aggregates assuming that initial platelet diameter was 3 μm (mono – 1); 3.8 μm (dimer – 2); 4.8 μm (tetra - 4); 6 μm (octa – 8); 7.6 μm (oligo – 16); 9.5 μm (oligo – 32); 12.0 μm (oligo – 64); 15.1 μm (oligo – 128); 19.0 μm (oligo – 256).

For these particles using the MiePlot software [3], we calculated and plotted the angle diagram considering that the number of particles at each step decreased two-fold (Supplementary Figure S4a). Based on the calculated angle diagram we estimated the corresponding changes in the LSI signal at different scattering angles during the increase of aggregate diameters, and the initial LSI signal was taken as 100% (Supplementary Figure S4b). According to our allotments, the LSI during aggregation should have increased at the scattering angles from 0.5° to 2° and decreased at the angles from 8 to 12°. Thus, platelet aggregation could be adequately characterized by the changes in the LSI signal registered at 1°. Data from our model are in good agreement with the experimental data presented in Figure 1 of the manuscript and Supplementary Figure S2.

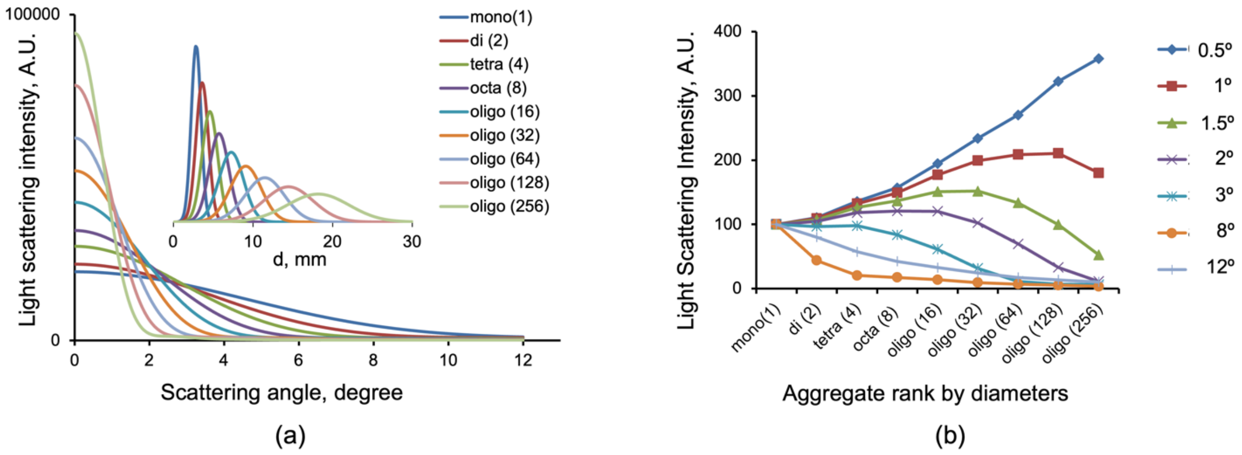

Supplementary Figure S4. Approximation of platelet aggregation according to Mie scattering simulation for analysis by laser diffraction method. **(a) –** Calculated angle diagram of light scattering for spherical particles of different diameters. **(b) –** The predicted LSI dynamics in the range of scattering angles from 0.5 to 12° during a sequential increase of aggregate diameters. The indicatrix corresponding to the "mono" rank was taken as 100%.

The data from Approximation (Supplementary Figure S4b) are in agreement with our experimental LSI traces (Supplementary Figure S2). Therefore, our data confirmed that laser diffraction could be used for aggregation description.

S2. Calibration of $[Ca^{2+}]_i$ measured by LaSca TMF

To calibrate intracellular Ca^{2+} concentration in platelets stained by Fluo-3 AM we used well-established calcium ionophore A23187 to equilibrate the extra- and intracellular calcium concentration. As soon as the Fluo-3 fluorescence intensity (FI) reached its maximum (continuous plateau of the FI signal), EGTA in overabundant concentration (16 mM) to bind all Ca^{2+} cations was applied and the decrease in FI was registered until it reached the minimum. Next, using Equation S1 and Fluo-3 Kd 390 nM, the $[Ca^{2+}]_i$ changes were calculated in dynamics (Supplementary Figure S5b). The initial $[Ca^{2+}]_i$ was estimated to be around 100 nM, which is in good agreement with the literature. These data confirmed that the fluorescence module of LaSca-TMF can be used for $[Ca^{2+}]_i$ assessment.

$$[Ca^{2+}]_i = K_d(F - F_{min})/(F_{max} - F) \qquad (S1),$$

where K_d is a dissociation constant for Ca^{2+} which is reported (according to the manufacturer) to be 390 nM;

F_{min} is the fluorescence intensity of the indicator in the absence of calcium;

F_{max} is the fluorescence of the calcium-saturated indicator;

and F is the fluorescence at intermediate calcium levels;

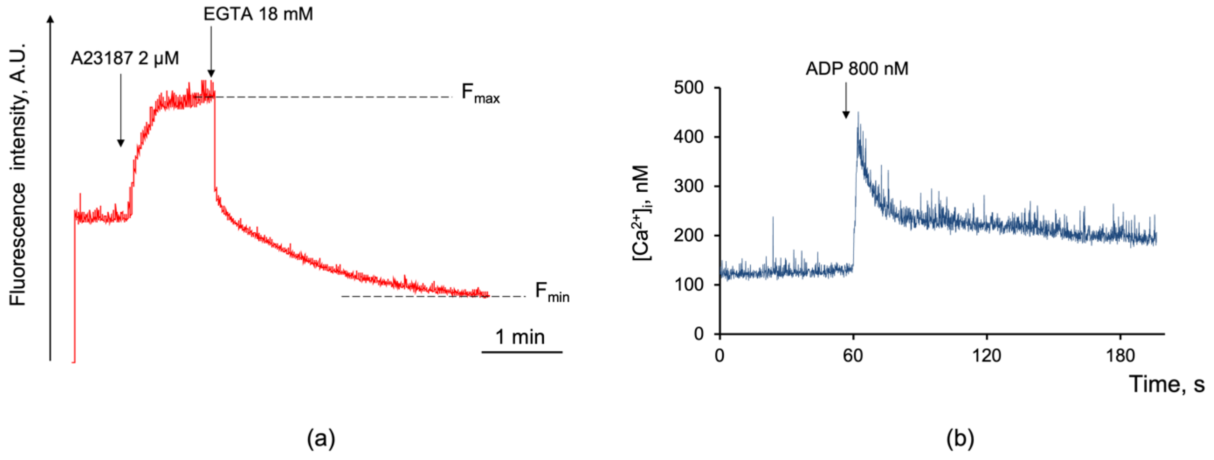

Supplementary Figure S5. Calibration of $[Ca^{2+}]_i$ in platelets. PRP was loaded with Fluo 3-AM (5 μM, 30 min, RT), diluted in HEPES buffer to a final concentration of 1 x 10^7 platelets/ml, and added to the cuvette with a continuous stirring (1200 rpm) at 25ºC. After the stabilization of the FI signal corresponding to the control cells (2 min), calcium ionophore A23187 (5 μM) was added to the platelet suspension. After maximal $[Ca^{2+}]_i$ was achieved, EGTA in overabundant concentration (16 mM) was added to bound the Ca^{2+} ions, and the signal was registered for an additional 5 min. Maximal $[Ca^{2+}]_i$ concentration is indicated as F_{max}, minimal, as F_{min}. **(a)** – The representative trace of FI signal of Fluo 3 measured at 529 nm; **(b) -** $[Ca^{2+}]_i$ changes after the ADP (800 nM) application registered in dynamics and calculated according to equation 1.

S3. Changes in platelet reactivity at 37 °C and 25 °C are independent of PKA/PKG activity

To evaluate whether the differences in platelet responses registered at 37°C and 25°C were associated with PKA/PKG activation, we analyzed VASP phosphorylation in platelets under these conditions (Supplementary

Figure S6). VASP is an established substrate for PKA and PKG and is commonly used as a marker of PKA/PKG kinase activation [4]. Phosphorylation of VASP at Ser-157 increases its apparent molecular mass in SDS/PAGE from 46 to 50 kDa and Ser-239 phosphorylation can be analyzed by phospho-specific antibody [5]. We evaluated VASP phosphorylation by flow cytometry and Western blot methods. Both methods did not demonstrate any changes in VASP phosphorylation associated with platelet incubation at different temperatures (Supplementary Figure S6).

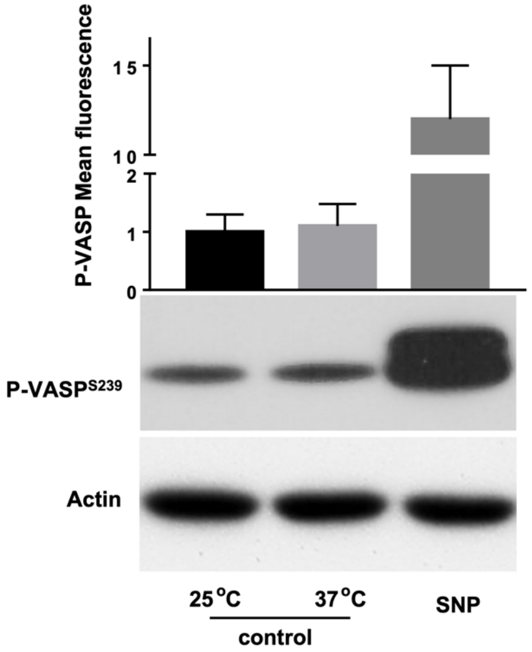

Supplementary Figure S6. Changes in platelet reactivity at 37 °C and 25 °C are independent of PKA/PKG activity. Diluted PRP (1 x 10^7 platelets/ml) was incubated in the cuvette with continuous stirring (1200 rpm) at 37 °C and 25 °C for 10 min or with SNP (5 μM, 2 min) as a positive control for PKA/PKG activation and subjected to VASP phosphorylation (P-VASP^{Ser239}) by Western blot or flow cytometry analysis. Actin blot was used as the loading control. Data on the graph are presented as Means ± SD, and P-VASP values at 25°C were taken as 1, n = 4. Shown is a representative Western blot out of three independent experiments.

S3.1 Methods for Supplementary Figure S6.

VASP phosphorylation analysis by flow cytometry. Diluted PRP (1 x 10^7 platelets/ml) was incubated in the cuvette at 37 °C and 25 °C for 10 min, then fixed with 2% formaldehyde for 10 min, and VASP^{Ser239} phosphorylation was analyzed according to [6]. Briefly, after fixation platelets were pelleted for 10 s at 8000 x g, resuspended in Triton-X100 (0.2%) in PBS, and permeabilized for 10 min. Next, platelets were incubated with P-VASP^{Ser239} antibody (clone 16C2, Nano-tools Teningen, Germany; final concentration of 2.4 μg/ml) for 30 min and centrifuged (1 min, 2700 x g). Platelet pellets were resuspended in PBS with FITC-conjugated goat anti-mouse antibodies (final concentration 25 μg/ml; Sigma, Germany) for 20 min at 4°C, washed with PBS, and analyzed by CytoFLEX flow cytometer.

VASP phosphorylation analysis by Western blot. Diluted PRP was incubated in the cuvette at 37 °C and 25 °C for 10 min, then platelets were pelleted by centrifugation (2.500 x g) for 3 min and lysed in Laemmly sample buffer. Proteins were separated by SDS/PAGE, transferred to a nitrocellulose membrane, blocked with milk

(3%) in Tris-buffered saline/0.1% Tween and incubated with primary antibodies against P- VASP^{Ser239} or actin overnight at 4°C. For visualization, goat anti-rabbit or anti-mouse IgG horseradish peroxidase conjugated were used as secondary antibodies followed by ECL detection (Amersham, Pharmacia Biotech). Anti-actin and secondary antibodies were from Cell Signaling (Frankfurt, Germany). For positive control in both experiments, platelets were incubated with SNP (5 µM, 2 min) and then processed for Western blot analysis.

LIST OF REFERENCES

1. Mindukshev, I., et al., *Low angle light scattering analysis: a novel quantitative method for functional characterization of human and murine platelet receptors.* Clin Chem Lab Med, 2012. **50**(7): p. 1253-62.
2. Affolter, H. and A. Pletscher, *Rheo-optical shape analysis of human blood platelets.* Thromb Haemost, 1982. **48**(2): p. 204-7.
3. Philip Laven. *MiePlot*. 2021, November 10 Available from: http://www.philiplaven.com/mieplot.htm.
4. Gambaryan, S., et al., *Potent inhibition of human platelets by cGMP analogs independent of cGMP-dependent protein kinase.* Blood, 2004. **103**(7): p. 2593-600.
5. Smolenski, A., et al., *Analysis and regulation of vasodilator-stimulated phosphoprotein serine 239 phosphorylation in vitro and in intact cells using a phosphospecific monoclonal antibody.* J Biol Chem, 1998. **273**(32): p. 20029-35.
6. Schwarz, U.R., et al., *Flow cytometry analysis of intracellular VASP phosphorylation for the assessment of activating and inhibitory signal transduction pathways in human platelets--definition and detection of ticlopidine/clopidogrel effects.* Thromb Haemost, 1999. **82**(3): p. 1145-52.
